# Predicting the Risk of Metastases by PSMA-PET/CT—Evaluation of 335 Men with Treatment-Naïve Prostate Carcinoma

**DOI:** 10.3390/cancers13071508

**Published:** 2021-03-25

**Authors:** Stefan A. Koerber, Johannes Boesch, Clemens Kratochwil, Ingmar Schlampp, Jonas Ristau, Erik Winter, Stefanie Zschaebitz, Luisa Hofer, Klaus Herfarth, Klaus Kopka, Tim Holland-Letz, Dirk Jaeger, Markus Hohenfellner, Uwe Haberkorn, Juergen Debus, Frederik L. Giesel

**Affiliations:** 1 Department of Radiation Oncology, Heidelberg University Hospital, 69120 Heidelberg, Germany; ingmar.schlampp@med.uni-heidelberg.de (I.S.); jonas.ristau@med.uni-heidelberg.de (J.R.); klaus.herfarth@med.uni-heidelberg.de (K.H.); Juergen.Debus@med.uni-heidelberg.de (J.D.); 2 National Center for Tumor diseases (NCT), Heidelberg University Hospital, 69120 Heidelberg, Germany; 3 Heidelberg Institute of Radiation Oncology (HIRO), 69120 Heidelberg, Germany; 4 Department of Nuclear Medicine, Heidelberg University Hospital, 69120 Heidelberg, Germany; j.boesch@gmx.de (J.B.); clemens.kratochwil@med.uni-heidelberg.de (C.K.); Erik.Winter@med.uni-heidelberg.de (E.W.); uwe.haberkorn@med.uni-heidelberg.de (U.H.); frederik.giesel@med.uni-heidelberg.de (F.L.G.); 5 Clinical Cooperation Unit Nuclear Medicine, German Cancer Research Center (DKFZ), 69120 Heidelberg, Germany; 6 Department of Medical Oncology, National Center for Tumor Diseases (NCT), Heidelberg University Hospital, 69120 Heidelberg, Germany; stefanie.zschaebitz@med.uni-heidelberg.de (S.Z.); dirk.jaeger@med.uni-heidelberg.de (D.J.); 7 Department of Urology, Heidelberg University Hospital, 69120 Heidelberg, Germany; Luisa.Hofer@med.uni-heidelberg.de (L.H.); markus.hohenfellner@med.uni-heidelberg.de (M.H.); 8 Heidelberg Ion-Beam Therapy Center (HIT), Department of Radiation Oncology, Heidelberg University Hospital, 69120 Heidelberg, Germany; 9 German Cancer Consortium (DKTK), Partner Site Dresden, 01328 Dresden, Germany; k.kopka@hzdr.de; 10 Helmholtz-Zentrum Dresden-Rossendorf, Institute of Radiopharmaceutical Cancer Research, 01328 Dresden, Germany; 11 Faculty of Chemistry and Food Chemistry, Technische Universität Dresden, 01069 Dresden, Germany; 12 Department of Biostatistics, German Cancer Research Center (DKFZ), 69120 Heidelberg, Germany; t.holland-letz@dkfz-heidelberg.de; 13 German Cancer Consortium (DKTK), Partner Site Heidelberg, 69120 Heidelberg, Germany; 14 Clinical Cooperation Unit Radiation Oncology, German Cancer Research Center (DKFZ), 69120 Heidelberg, Germany

**Keywords:** prostate cancer, PSMA, PET, metastases, intraprostatic SUV

## Abstract

**Simple Summary:**

Prostate carcinoma is the most common visceral cancer for men and the second most common cause of death. The early detection of micrometastasis may improve clinical outcome due to individual treatment approaches like early intensified therapy. Imaging using prostate-specific membrane antigen-positron emission tomography/computed tomography (PSMA-PET/CT) has a high potential of detecting even small metastases. Therefore, the present study aimed to analyze data of 335 men with primary diagnosed prostate cancer and available PSMA-PET/CT with regard to characteristic PET-parameters and the detection of metastases. We observed that an increased accumulation of the PET-tracer measured in the primary tumor significantly correlates with the presence of distant metastases. The current results may be helpful in decision making of individual treatment escalation for a variety of men with aggressive disease which should improve clinical outcome.

**Abstract:**

Men diagnosed with aggressive prostate cancer are at high risk of local relapse or systemic progression after definitive treatment. Treatment intensification is highly needed for that patient cohort; however, no relevant stratification tool has been implemented into the clinical work routine so far. Therefore, the aim of the current study was to analyze the role of initial PSMA-PET/CT as a prediction tool for metastases. In total, 335 men with biopsy-proven prostate carcinoma and PSMA-PET/CT for primary staging were enrolled in the present, retrospective study. The number and site of metastases were analyzed and correlated with the maximum standardized uptake value (SUVmax) of the intraprostatic, malignant lesion. Receiver operating characteristic (ROC) curves were used to determine sensitivity and specificity and a model was created using multiple logistic regression. PSMA-PET/CT detected 171 metastases with PSMA-uptake in 82 patients. A statistically significant higher SUVmax was found for men with metastatic disease than for the cohort without distant metastases (median 16.1 vs. 11.2; *p* < 0.001). The area under the curve (AUC) in regard to predicting the presence of any metastases was 0.65. Choosing a cut-off value of 11.9 for SUVmax, a sensitivity and specificity (factor 1:1) of 76.0% and 58.4% was obtained. The current study confirms, that initial PSMA-PET/CT is able to detect a relatively high number of treatment-naïve men with metastatic prostate carcinoma. Intraprostatic SUVmax seems to be a promising parameter for the prediction of distant disease and could be used for treatment stratification—aspects which should be verified within prospective trials.

## 1. Introduction

With an estimated 1.93 million deaths from cancer in Europe in 2018 [1], the treatment of patients with tumor diseases is still demanding. For men, carcinoma of the prostate is the most common visceral cancer with estimated 449,800 new cases in 2018 [1]. Although many cases of prostate cancer have excellent prognosis or stay indolent for a lifetime, the malignant tumor remains a heterogeneous disease varying from subclinical to highly aggressive. 

Therefore risk-adopted diagnosis and treatment of prostate cancer is challenging to avoid overtreatment for indolent disease and insufficient therapy for carcinomas with a high risk for local or systemic progression. By current estimates, high-risk disease is diagnosed in up to 20% of patients undergoing definitive treatment [2,3,4,5]. Active and highly effective therapy is of utmost necessity for this patient cohort. However, for high-risk prostate cancer local progression and/or distant metastases were observed in 11–40% after definitive radiotherapy or radical prostatectomy, respectively [6,7,8]. These patients are candidates for treatment intensification to prolong survival. 

The RTOG 0521 trial included 612 men with high risk prostate cancer undergoing definitive radiotherapy with long-term androgen deprivation therapy (ADT). The trial resulted in improved overall survival (OS), disease-free survival (DFS) and reduction in the rate of distant metastasis for the cohort with intensified systemic therapy with adjuvant docetaxel after a median follow-up of 5.7 years [8]. In contrast, two Scandinavian trials did not observe any relevant, clinical benefit by adding chemotherapy. Both, the Scandinavian Prostate Cancer Group (SPCG)-12 and -13, were not able to show a significant benefit in DFS by adding adjuvant chemotherapy [9,10]. These differences clearly demonstrate, that there is an urgent need for an appropriate risk classification which is able to identify candidates for treatment intensification. 

Nowadays, prostate-specific membrane antigen (PSMA)-positron emission tomography/computed tomography (PET/CT) is an important element of prostate carcinoma imaging. PSMA is an extracellular, membrane-bound antigen that is not secreted, but is overexpressed on almost all prostate tumors [11,12,13,14]. In only about 5–10% of all prostate carcinomas there is no PSMA expression [15,16]. By using radioactively labeled substances that specifically bind to PSMA, malignant prostate cells which excessively express PSMA can be detected with a sensitivity of 76.6–84% and a specificity of 82–100% [17,18,19,20]. The majority of the low molecular weight PSMA ligands are based on a glutamate-urea-lysine structure and two other units [17]. The radioactive unit is used for imaging and consists of a chelator molecule, which forms a covalent bond with a radioactive molecule (as in 68Ga) or from a group of fluorinated molecules (as in 18F) [18]. A chelator serves to stabilize the radioactive molecule via a covalent bond by fixing it [19]. The radioactive unit in turn is coupled via a connector to a binding group which can bind in the active center of the PSMA molecule. The binding motif best described in science consists of the combination glutamate-urea-lysine (Glu-urea-Lys) [18].

Currently, two groups of 68Ga-coupled PSMA ligands (such as ^68^Ga-PSMA-11, ^68^Ga-PSMA-I + T and ^68^Ga-PSMA-617) and ^18^F-coupled ligands (such as ^18^F-DCPyL, ^18^F-PSMA-1007) are used world wide. While PSMA-PET/CT imaging was mainly used for patients with biochemical recurrence for a long time, the number of PET/CTs using PSMA-ligands as a primary imaging probe rapidly increased. Results of a recently published, randomized prospective study clearly underlined its importance also for first-line staging of prostate cancer patients before curative-intent surgery or radiotherapy [20]. Therefore, the current study aimed to evaluate the role of PSMA-PET/CT for the prediction of metastases.

## 2. Materials and Methods

### 2.1. Study Design

This monocentric and retrospective study, which was approved by the local ethics committee (S-093/2019) and conducted in accordance with the Helsinki Declaration and its amendments. This present study enhances and analysis new aspects of a patient cohort partially previously reported [21]. In total, 335 men with histologically proven, treatment-naïve prostate cancer met the inclusion criteria (age of 18 or older, sufficient clinical data, no previous therapy like ADT, high-intensity focused ultrasound (HIFU) or transurethral resection of the prostate (TURP)) and were included in the current trial. 

### 2.2. Prostate-Specific Membrane Antigen-Positron Emission Tomography/Computed Tomography (PSMA PET/CT) Imaging and Image Evaluation

All patients were hydrated with 500 mL water two hours before imaging. Imaging was performed by Siemens mCT flow, Siemens Biograph 6 and Siemens Biograph 20 mCT scanners. Low-dose CTs without contrast were performed for all patients (5 mm slices). For ^68^Ga-PSMA-11, two to three MBq per kilogram body weight were applied and the images were taken according to the first clinical description one hour after application [22]. The median uptake time was 60 min between application and acquisition. For ^18^F-PSMA-1007, an effective dose of approximately 4.4–5.5 mSv per 200–250 MBq examination was applied [23]. The median dose of ^68^Ga-PSMA-11 was 223 MBq and 254 MBq for ^18^F-PSMA-1007, respectively. Imaging acquisition was performed after 60 min for ^68^Ga-PSMA-11 and 90–120 min for ^18^F-PSMA-1007 after application [24]. 

Image evaluation was done using the “syngo TrueD” software (Siemens Healthineers, Erlangen, Germany). The subsequent determination of the maximum standardized uptake values (SUVmax) was performed via a semi-automatic region of interest (ROI). All scans were evaluated by two certified nuclear physicians as well as one certified radiooncologist. Any tracer accumulation larger than 3 mm diameter that did not correspond to a physiological uptake and was observed outside the prostate was considered primarily tumor. All these findings were collected in a common consensus. Due to several publications [18,21,24,25] a direct correlation of histopathology and PSMA-imaging findings has been assumed in this study.

### 2.3. Statistical Analysis

Statistical analysis was performed using Microsoft Excel and SPSS Statistics version 26 (IBM, Armonk, NY, USA). The descriptive analysis of the SUVmax values between patients with and without distant metastases was displayed using box plots. Univariable and multivariable binary logistic regression models were calculated. The dependent variable in all cases was: metastasis occurred yes or no. Univariable models were calculated for four variables, SUVmax (continuous), initial prostate-specific-antigen (PSA) (continuous), World Health Organization (WHO) grading (categorical, 5 classes) and d’Amico classification (categorical, 3 classes). The multivariable model includes all four variables [25]. Results are expressed as odds ratios with 95% confidence intervals for each variable, as well as the area under the curve (AUC) statistic for the whole model. These receiver operating characteristic (ROC) curves were used to determine sensitivity and specificity as a function of the association between the binary characteristic “any metastasis occurred” and the quantitative characteristic “SUVmax”. From the variables of the multiple logistic regression model, the variable “probability of occurrence p” could be determined by transforming the regression equation. The resulting variable “probability of occurrence p” could again be examined in terms of a ROC curve (Receiver Operating Characteristic) in relation to the binary variable of “metastasis that has occurred”. The area under the curve (AUC) values were again determined on the basis of the curve thus calculated.

## 3. Results

### 3.1. Cohort Characteristics

The cohort included 335 men with histologically confirmed prostate carcinoma after biopsy. All patients had no previous therapy. Median age of the entire cohort was 67 years (range 38–84 years) and a median PSA of 11 ng/mL (range 1.2–511 ng/mL) was found at initial diagnosis. Histological examinations resulted in 148 patients (44.2%) with WHO grading 4 or 5 carcinomas. Most patients (65.4%) were classified as high-risk according to the d’Amico risk classification [16]. Patient’s characteristics are summarized in Table 1. 

### 3.2. Imaging Evaluation

PSMA imaging detected 81 patients (24.2%) with regional nodal metastases. In total, 82 patients (24.7%) with 171 distant, PSMA-positive lesions were diagnosed. Most metastases were located in extrapelvic nodes (*n* = 60) and bone (*n* = 103), the rate of visceral metastases was low with eight lesions with tracer uptake (Table 2). Median SUVmax of malignant intraprostatic lesions was 12.5 (range 3–109) for the entire cohort. 48 out of 82 patients with distant metastases also had regional, nodal metastases with PSMA-uptake. 

### 3.3. Association between PSMA-PET/CT and the Presence of Metastases

**Figure 1:**
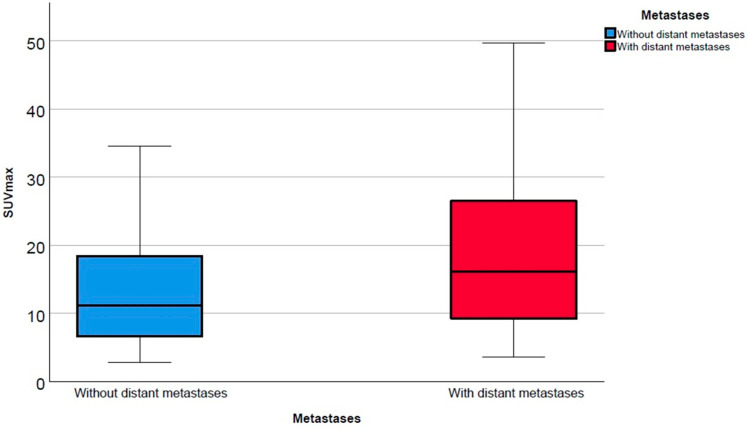
Boxplot of intraprostatic maximum standardized uptake values (SUVmax) according to M-stage.

Correlating the presence of metastases and intraprostatic SUVmax in PSMA imaging, a higher SUVmax was observed for men with metastatic disease than for those without metastases (median 11.2 vs. 16.1; *p* < 0.001) (Figure 1). 

The AUC in regard to predicting the presence of metastases was 0.65. In subgroup analyses, the best AUC was obtained for patients with intermediate-risk prostate cancer (0.71; low-risk: 0.29; high-risk: 0.56) and after exclusion of International Society of Urological Pathology (ISUP) grade group 5 (0.66). Moreover, the AUC was significantly better for PET scans using 18F-PSMA ligands compared to those with 68Ga-PSMA ligands (0.71 vs. 0.63—Supplementary File 1 and Supplementary File 2).

Comparing different combinations of intraprostatic SUVmax, PSA, WHO grading and d’Amico risk classification in a multiple binary logistic regression model, all variables showed a high, statistical significance with regard to distant metastases (*p* < 0.001). Multiple logistic regression model calculations of all parameters lead to a slightly better AUC of 0.69 (Table 3). The multiple logistic regression showed a highly significant (*p* = 0.004) influence of SUVmax on the occurrence of distant metastasis. 

The maximum for the sum of sensitivity and specificity (weight factor 1:1) was observed using a cut-off value of 11.9 for SUVmax of malignant intraprostatic lesions, resulting in a sensitivity and specificity of 76.0% and 58.4%, respectively. Assuming a prevalence of 24.5% for metastases, the negative predictive value (NPV) and positive predictive value (PPV) could be stated as 85.3% and 33.1%.

## 4. Discussion

The present study confirmed, that a relatively large number of men with prostate cancer are at high risk for metastases. Distant, malignant lesions occurred in almost one in four patients in the current cohort, even though, considering the “negative” selection of men undergoing PSMA-PET/CT as primary staging (219 out of 335 with high-risk disease). However, while 5-year survival rates are excellent for localized prostate cancer, lifespan is limited for patients with distant tumor burden. Siegel et al. reported on a 5-year relative survival rate of 31% in men with metastatic disease [26]. Thus, treatment intensification strategies are of great interest for selected prostate cancer patients. 

**Figure 2:**
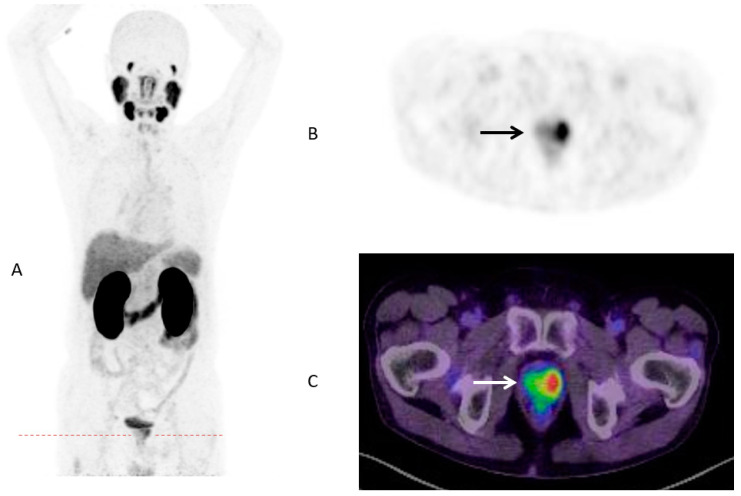
PSMA-PET/CT in maximum intensity projection (MIP) (**A**) of a 70-years old patient with prostate cancer (GS 7b/group grade 3, PSA 4.53 ng/mL) and low intraprostatic SUVmax (3.45) and no metastases (**B**) PSMA-PET Dx; (**C**) PSMA PET/CT Dx.

**Figure 3:**
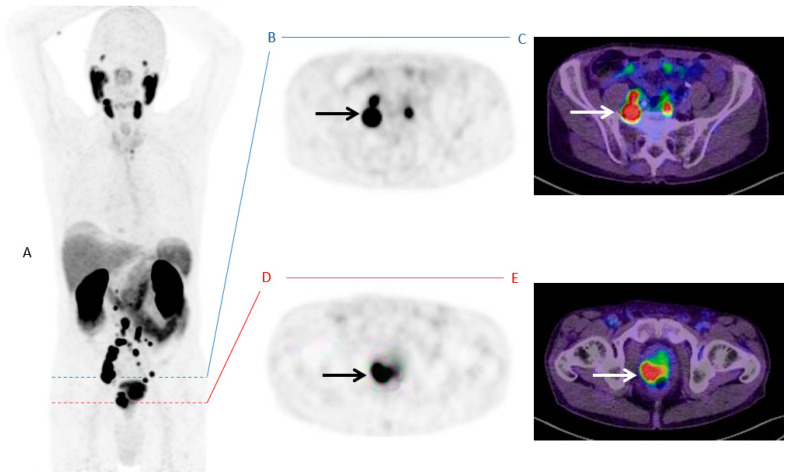
PSMA-PET/CT in MIP (**A**) of a 83-years old patient with prostate cancer (GS 8/group grade 4; PSA 32 ng/mL) and high intraprostatic SUVmax (49.63) and several metastases; Level of prostate (**B**) PSMA-PET Dx; (**C**) PSMA PET/CT Dx; Level of nodal metastases (**D**) PSMA-PET Dx; (**E**) PSMA PET/CT Dx.

Until now, the identification of patient subgroups suitable for treatment escalation leading to improved survival is still challenging. Although numerous pretreatment risk stratification tools like the Memorial Sloan Kettering Cancer Center (MSKCC) nomogram or the Cancer of the Prostate Risk Assessment (CAPRA) score are available [27], there is a lack of evident data with regard to individual therapeutic consequences. Increasing numbers of PSMA-PET/CTs for primary staging around the globe confirms a great potential to that molecular imaging methodology. As published a few years ago, SUVmax of intraprostatic, malignant lesions was highly correlated with several clinical parameters like Gleason Score (GS) or PSA [28]. The present study observed a statistically significant higher SUVmax for men with metastatic disease (Figure 2 and Figure 3). 

Choosing a cut-off value of 11.9, a relatively high sensitivity and specificity can be reached with regard to the presence of metastases leading to an estimated NPV and PPV of 85.3% and 33.1%, respectively, for the current cohort. Although subgroup analysis demonstrated a higher correlation for patients undergoing ^18^F-PSMA-1007 PET/CT compared with ^68^Ga-PSMA11-PET/CT, this cut-off value was determined for both tracers considering the relatively low number of patients who received ^18^F-PSMA-1007. A collection of two separate cut-off values did not seem sensible in this context. Nevertheless, the standard-PSA-blood test for prostate cancer screening provides a NPV and PPV of 15.5% and 25%, respectively [29], which is considerably lower compared to the current results. Thus, our findings suggest a high potential for PSMA-PET/CT predicting metastasis which might be integrated in clinical decision making for individual treatment escalation. In support of this hypothesis Komek et al. reported an association between SUV parameters from PSMA-imaging and survival outcome. In a cohort of 148 men with advanced prostate cancer undergoing ^68^Ga-PSMA-PET/CT, several SUV parameters like bone SUV >10.7 were significant in the determination of increased mortality risk [30]. Hence, SUV from PSMA-PET/CT seems to be a promising parameter for prostate cancer risk stratification in daily clinical practice. 

In this context it is interesting to note, that for the present trial a stronger correlation was obtained when using ^18^F-PSMA-1007 compared with ^68^Ga-PSMA-11 (both ROC curves are depicted in the Supplementary File 2). Due to the retrospective nature of the current study including patients who have been investigated over a longer period, the ^68^Ga-PSMA-11-tracer was initially more widespread. Over time, the 18F-PSMA-tracer was later introduced and more frequently utilized at the Department of Nuclear Medicine due to improved biological properties explaining the analysis of both tracers. This leads to the fact that both subgroups were not well-balanced. However, similar differences were observed in literature: a head-to-head comparison of ^68^Ga-PSMA-11 and ^18^F-PSMA-1007 resulted in excellent identification of all dominant factors.

Prostatic lesions for both tracers, however, SUVmax was subject to significant variations depending on tracer type. For almost all patients, SUVmax obtained by ^18^F-PSMA-1007 was significantly higher than by ^68^Ga-PSMA-11 (factor 1.1–3.9) [31].

Even though to the best of our knowledge the present study is the first to evaluate the correlation of SUVmax of intraprostatic malignant lesion and the prediction of metastases, some limitations need to be highlighted. Although the number of patients included in the current study was relatively high, the trial had a retrospective design and was performed at only one institution. This makes it prone for patient selection biases only to be overcome by prospective trials. Moreover, the size of the individual metastases was not explicitly recorded and confounders like inflammation of the prostate may influence measurements of SUVmax affecting the interpretation of hybrid imaging results [32]. Although the current study did not include a correlation with histopathological data, the conclusions seem to be legitimate. Many analyses have already focused on the correlation of PSMA-PET/CT and histopathological data. In a cohort of 319 men who underwent ^68^Ga-PSMA-11 PET/CT after PSA relapse, PSMA-imaging demonstrated excellent histopathological correlation [33]. Moreover, a sensitivity of 94.7% for nodal disease using ^18^F-PSMA-1007 for primary staging was also observed [23]. Several other trials confirmed these observations after correlation of data obtained by PSMA-PET/CT and results from surgery [34,35]. Sensitivity and specificity ranged from 75–99% for both. Due to strong published results, a direct correlation of histopathology and PSMA-imaging findings was assumed in the current analysis.

In summary, the present study demonstrated promising results and provides an excellent basis for prospective trials evaluating the integration of PSMA-PET/CT as a stratification tool for prostate cancer patients with a high risk for systemic disease. Further research is required to assess the role of different tracers and evaluate the impact on survival outcome obtained by PSMA-guided treatment intensification.

## 5. Conclusions

The present study confirms, that there is a high rate of treatment-naïve prostate cancer patients with distant disease identified by PSMA-PET/CT. Including SUVmax of malignant, intraprostatic lesions, may be helpful in decision making of individual treatment escalation for a variety of men with aggressive disease which should improve clinical outcomes.

## Figures and Tables

**Table 1:** Patient’s characteristics.

Total Number of Patients	*n =* 335
age at PSMA-PET/CT (years), median (range)	67 (38–84)
WHO grading, *n =* 326	
1	36 (10.7%)
2	85 (25.4%)
3	57 (17.0%)
4	58 (17.3%)
5	90 (26.9%)
PSA at initial diagnosis (ng/mL), median (range), *n =* 331	11 (1.2–511)
<10	153 (45.7%)
10–20	84 (25.1%)
>20	94 (28.1%)
risk classification according d’Amico, *n =* 335	
low risk	15 (4.5%)
intermediate risk	101 (30.1%)
high risk	219 (65.4%)
PSMA-PET/CT tracer	
^68^Ga-PSMA-11	272 (81.2%)
^18^F-PSMA-1007	63 (18.8%)
N staging according to PSMA-PET/CT, *n =* 335	
cN0	254 (75.8%)
cN1	81 (24.2%)
M staging according to PSMA-PET/CT, *n =* 335	
cM0	253 (75.5%)
cM1a	18 (5.4%)
cM1b	39 (11.6%)
cM1c	25(7.5%)

**Table 2:** Overview of site of metastases detected by PSMA-PET/CT.

Total Number of Distant Metastases with PSMA-Uptake	*n =* 173
extrapelvic nodal metastases	
total lesions	60 (35.1%)
abdominal	42 (70.0%)
Thoracic	15 (25.0%)
Cervical	1 (1.7%)
others	2 (3.3%)
bone metastases	
total lesions	103 (60.2%)
Pelvic	43 (41.7%)
abdominal/thoracic	49 (47.6%)
Extremities	8 (7.8%)
head/cervical	3 (2.9%)
organ metastases	
total lesions	10 (5.7%)
Intrahepatic	4 (40.0%)
Pulmonal	4 (40.0%)
others	2 (20.0%)

**Table 3:** Multiple logistic regression.

Variables	-	Regression Coefficient B	Standard Error	*p*-Value	Odds Ratio	95% Confidence Intervals for Odds Ratio	
-	-	-	-	-	-	Lower value	Upper value
SUVMax	-	0.026	0.009	0.004	1.026	1.006	1.046
initial PSA	-	0.008	0.004	0.051	1.008	1.000	1.016
WHO ^1^	-	-	-	0.264	-	-	-
-	WHO (1)	−1.374	0.819	0.093	0.253	0.054	1.344
-	WHO (2)	−0.798	0.456	0.316	0.639	0.200	1.191
-	WHO (3)	−0.448	0.446	0.316	0.639	0.252	1.479
-	WHO (4)	−0.510	0.383	0.183	0.601	0.288	1.292
d’Amico ^1^	-	-	-	0.476	-	-	-
-	d’Amico (1)	−0,332	0.064	0.800	0.717	0.054	9.094
-	d’Amico (2)	−0.559	0.459	0.224	0.572	0.204	1.280

^1^ Reference categories have been “WHO5” and “d’Amico high-risk.

## Data Availability

The data used and/or analyzed during the current study are available from the corresponding author on reasonable request.

## Supplementary Materials

The following are available online at https://www.mdpi.com/2072-6694/13/7/1508/s1, File 1: Univariate analysis of binary logistic regression SUVmax, WHO, PSA, d’Amico-Score; File 2: ROC curves of 68Ga and 18F.
