# Supplementary material for: Predicting the Risk of Metastases by PSMA-PET/CT—Evaluation of 335 Men with Treatment-Naïve Prostate Carcinoma
Source: Cancers (Basel). 2021 Mar 25;13(7):1508. doi: 10.3390/cancers13071508 (PMC8037082; doi:10.3390/cancers13071508)
Supplement: Supplementary file 1 [file cancers-13-01508-s001.zip › Table-4-7_Supplementary_file_1_Univariate-Analysis.docx]

Supplementary File #1: Univariate Analysis

*Table 4: Univariate analysis of binary logistic regression SUVmax*

|  |  |  |  |  | **95% Confidence intervals for odds ratio** | |
| --- | --- | --- | --- | --- | --- | --- |
| Variable | regression coefficient B | standard error | p-value | odds ratio | Lower valuer | Upper value |
| SUVmax | 0,34 | 0,009 | 0,000 | 1,035 | 1,016 | 1,054 |
| Constant | -1,730 | 0,207 | 0,000 | 0,177 |  |  |

*Table 5: Univariate analysis of binary logistic regression of WHO*

|  |  |  |  |  | **95% Confidence intervals for odds ratio** | |
| --- | --- | --- | --- | --- | --- | --- |
| Variable | regression coefficient B | standard error | p-value | odds ratio | Lower value | Upper value |
| WHO^1^ |  |  | 0,000 |  |  |  |
| WHO (1) | -1,899 | 0,641 | 0,003 | 0,150 | 0,043 | 0,526 |
| WHO (2) | -1,307 | 0,380 | 0,001 | 0,271 | 0,129 | 0,570 |
| WHO (3) | -0,720 | 0,383 | 0,060 | 0,487 | 0,204 | 0,945 |
| WHO (4) | -0,381 | 0,361 | 0,291 | 0,683 | 0,336 | 1,386 |
| Constant | -0,499 | 0,217 | 0,022 | 0,607 |  |  |

^1^ Reference categories has been “WHO5”

*Table 6: Univariate analysis of binary logistic regression of PSA_initial*

|  |  |  |  |  | **95% Confidence intervals for odds ratio** | |
| --- | --- | --- | --- | --- | --- | --- |
| Variable | regression coefficient B | standard error | p-value | odds ratio | Lower value | Upper value |
| PSA_initial | 0,012 | 0,004 | 0,002 | 1,012 | 1,004 | 1,020 |
| Constant | -1,414 | 0,160 | 0,000 | 0,243 |  |  |

*Table 7: Univariate analysis of binary logistic regression of D’Amico-Score*

|  |  |  |  |  | **95% Confidence intervals for odds ratio** | |
| --- | --- | --- | --- | --- | --- | --- |
| Variable | regression coefficient B | standard error | p-value | odds ratio | Lower value | Upper value |
| d’Amico^1^ |  |  | 0,000 |  |  |  |
| d’Amico (1) | -1,884 | 1,045 | 0,072 | 0,152 | 0,020 | 1,179 |
| d’Amico (2) | -1,346 | 0,351 | 0,000 | 0,260 | 0,115 | 0,477 |
| Constant | -0,755 | 0,145 | 0,000 | 0,470 |  |  |

1 Reference categories has been “d’Amico high-risk
