# Supplementary material for: Predicting the Risk of Metastases by PSMA-PET/CT—Evaluation of 335 Men with Treatment-Naïve Prostate Carcinoma
Source: Cancers (Basel). 2021 Mar 25;13(7):1508. doi: 10.3390/cancers13071508 (PMC8037082; doi:10.3390/cancers13071508)
Supplement: Supplementary file 1 [file cancers-13-01508-s001.zip › Table-8_Supplementary-File-2_ROC-curve-of-68Ga-and-18F.docx]

**Supplementary File #2: ROC curves of 68Ga and 18F**

**Table 8: ROC Curve analysis by use of Tracer**

| **Tracer** |  | **With distant metastases** | **Without distant metastases** | **AUC** |
| --- | --- | --- | --- | --- |
| 68Ga |  | 64 | 208 | 0,629 |
| 18F |  | 18 | 45 | 0,709 |

1- specificity

Sensitivity


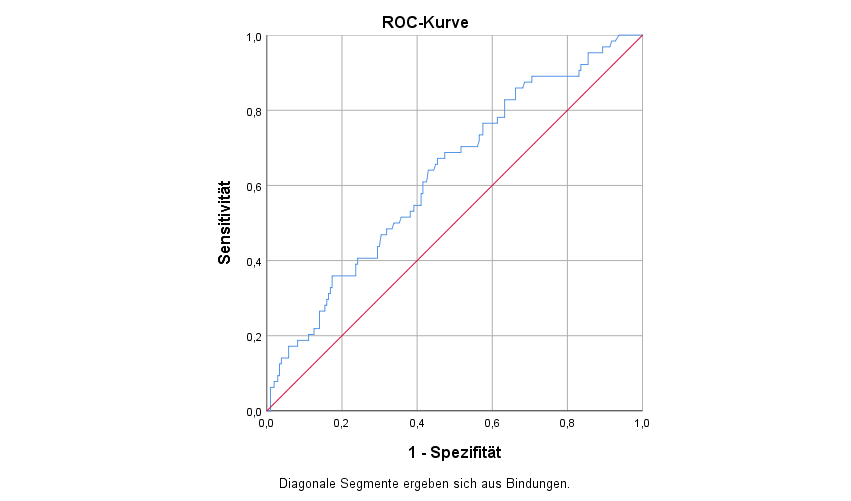

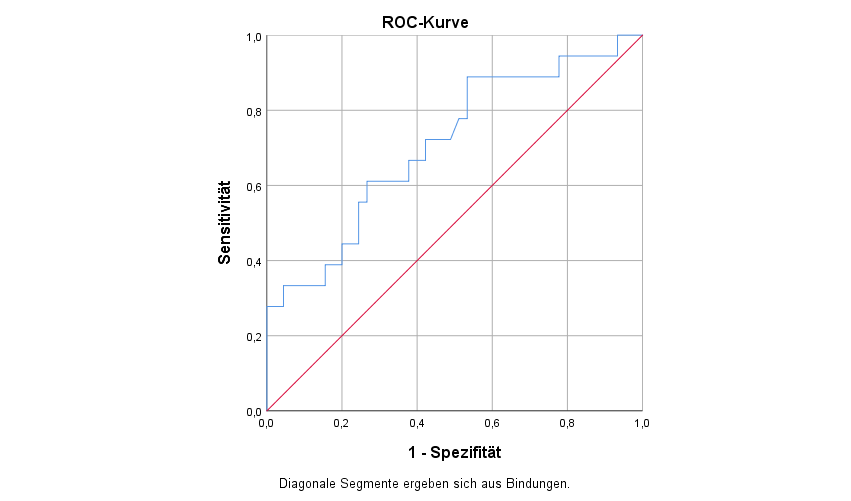


1- specificity

*Figure 4:*

*Left ROC Curve: ROC curve analysis: use of Tracer 68Ga: examination of correlation between SUVmax to distant metastasis [y = sensitivity; x = 1- specificity; AUC = 0,629]*

*Right ROC Curve: ROC curve analysis: use of Tracer 18F: examination of correlation between SUVmax to distant metastasis [y = sensitivity; x = 1- specificity; AUC = 0,709]*
